# Do Lessons in Nature Boost Subsequent Classroom Engagement? Refueling Students in Flight

**DOI:** 10.3389/fpsyg.2017.02253

**Published:** 2018-01-04

**Authors:** Ming Kuo, Matthew H. E. M. Browning, Milbert L. Penner

**Affiliations:** ^1^Natural Resources and Environmental Sciences, University of Illinois at Urbana–Champaign, Champaign, IL, United States; ^2^Recreation, Sport and Tourism, University of Illinois at Urbana–Champaign, Champaign, IL, United States; ^3^Cold Spring Environmental Studies Magnet School, Indianapolis Public Schools, Indianapolis, IN, United States

**Keywords:** classroom engagement, academic achievement, teaching outdoors, lessons in nature, environmental education

## Abstract

Teachers wishing to offer lessons in nature may hold back for fear of leaving students keyed up and unable to concentrate in subsequent, indoor lessons. This study tested the hypothesis that lessons in nature have positive—not negative—aftereffects on subsequent classroom engagement. Using carefully matched pairs of lessons (one in a relatively natural outdoor setting and one indoors), we observed subsequent classroom engagement during an indoor instructional period, replicating these comparisons over 10 different topics and weeks in the school year, in each of two third grade classrooms. Pairs were roughly balanced in how often the outdoor lesson preceded or followed the classroom lesson. Classroom engagement was significantly better after lessons in nature than after their matched counterparts for four of the five measures developed for this study: teacher ratings; third-party tallies of “redirects” (the number of times the teacher stopped instruction to direct student attention back onto the task at hand); independent, photo-based ratings made blind to condition; and a composite index each showed a nature advantage; student ratings did not. This nature advantage held across different teachers and held equally over the initial and final 5 weeks of lessons. And the magnitude of the advantage was large. In 48 out of 100 paired comparisons, the nature lesson was a full standard deviation better than its classroom counterpart; in 20 of the 48, the nature lesson was over two standard deviations better. The rate of “redirects” was cut almost in half after a lesson in nature, allowing teachers to teach for longer periods uninterrupted. Because the pairs of lessons were matched on teacher, class (students and classroom), topic, teaching style, week of the semester, and time of day, the advantage of the nature-based lessons could not be attributed to any of these factors. It appears that, far from leaving students too keyed up to concentrate afterward, lessons in nature may actually leave students more able to engage in the next lesson, even as students are also learning the material at hand. Such “refueling in flight” argues for including more lessons in nature in formal education.

## Introduction

When teachers offer lessons in relatively natural settings, students may benefit in a number of important ways. Academically, some evidence suggests students retain more after lessons in nature in biology and math (Fägerstam and Blom, 2012), language arts, social studies, and science more generally (Lieberman and Hoody, 1998) than after similar lessons indoors. Lessons in nature may also offer other benefits associated with exposure to trees, gardens, parks, and wildlife, including physical activity, stress relief, and the rejuvenation of attention (for reviews see Chawla, 2015; Kuo, 2015). Furthermore, as anthropogenic climate change becomes an increasingly pressing issue, lessons in nature may help build the next generation of environmental stewards; positive childhood nature experiences appear to play a key role in fostering pro-environmental behavior in adulthood (Monroe, 2003).

Perhaps in response to these important potential benefits, many European countries are incorporating lessons in nature in their formal schooling (Bentsen and Jensen, 2012); in the U.S., however, there has been relatively little embrace of outdoor formal instruction beyond the preschool setting (Ernst and Tornabene, 2012). One reason lessons in nature have not caught on in the U.S. may be a concern on the part of teachers that outdoor lessons will leave students keyed up and unable to concentrate. In the context of high-stakes testing, even temporary losses in classroom engagement are an important concern. Classroom engagement—the extent to which students are on-task and paying attention to the material or activity at hand—is both easily disrupted and a major driver of learning and academic success (Godwin et al., 2016). If lessons in nature do leave kids “keyed up” and unable to focus afterwards, then the benefits of that time may be outweighed by the costs.

Do lessons in nature impair subsequent classroom engagement? Our review of the environmental psychology literature suggests quite the opposite. Although we found no studies directly addressing this question, the indirect evidence suggests that classroom engagement will be enhanced, not impaired, immediately after lessons in nature. Specifically, spending time in relatively natural outdoor settings has a number of positive, immediate aftereffects on individuals, each of which is likely to enhance classroom engagement. Moreover, multiple studies have found that schools with greener, more vegetated surroundings perform better academically—even when socioeconomic factors are taken into account (Kuo et al., in review). Here we review the evidence on acute doses of contact with nature and their effects on cognitive functioning, interest in learning, and stress, as well as the literature tying greener schools with better academic achievement.

Attention is an important resource in student engagement (Pekrun and Linnenbrink-Garcia, 2012). Acute doses of nature, whether through a window view of a tree-lined street or a walk in a park, have positive aftereffects on attention and working memory. Attention restoration theory suggests that natural landscapes are gently absorbing, inducing a state of “soft fascination” that allows the mental muscle underlying our ability to deliberately direct attention to rest; afterwards, our capacity to direct attention is thereby refreshed (Kaplan, 1995; for a recent review of empirical work on attention restoration theory, see Ohly et al., 2016). Experimental work has demonstrated these aftereffects for classroom window views of greenery vs. barren schoolyards (Li and Sullivan, 2016), and for walks in both forested (van den Berg et al., 2017) and relatively green urban settings (Faber Taylor and Kuo, 2009) as compared to walks in less green urban settings. Thus, both a lesson in a relatively green spot in a schoolyard and the walks between that spot and the classroom might rejuvenate students' attention, enhancing their ability to concentrate on the next, indoor lesson.

Motivation is a similarly important resource in student engagement (Deci et al., 2011), and nature-based learning has been tied to high levels of engagement and enjoyment in a number of studies. Although we found no studies examining aftereffects of acute doses of nature, children prefer and enjoy lessons outdoors over lessons indoors (Mygind, 2009; Wistoft, 2013), and there is some indication that outdoor nature-based learning fosters greater interest in school and learning generally (e.g., Ernst and Stanek, 2006). Importantly, these effects may be largest in precisely the students whose motivation in “normal” classes is most lacking (Dettweiler et al., 2015). Nature-based learning appears to foster students' intrinsic motivation (Fägerstam and Blom, 2012; Skinner et al., 2012). Collectively, this body of work suggests nature-based instruction makes learning more interesting and enjoyable; might the interest and positive affect from a lesson in nature carry over to the next, indoor lesson, resulting in greater classroom engagement?

Stress is likely to be another important (negative) factor in student engagement; high levels of stress consistently predict lower levels of academic achievement (e.g., Grannis, 1992; Leppink et al., 2016). Experimental work in adults with physiological indicators shows that contact with nature offers quick and powerful reductions in stress biomarkers (e.g., Park et al., 2010; for review, see Kuo, 2015; Supplementary Materials), and this effect appears to extend to children as well. Contact with nature has been tied to lower levels of both self-reported and physiological measures of stress in multiple studies with children (Bell and Dyment, 2008; Chawla, 2015; Wiens et al., 2016). Recently an experimental study involving high school students showed that even a mere window view of vegetation from a classroom yields systematic decreases in both heart rate and self-reported stress, whereas a classroom without windows does not (Li and Sullivan, 2016). Further, students learning in a forest setting one day a week showed healthier diurnal rhythms in the stress hormone cortisol in that setting than a comparison group that did not receive outdoor learning—and these effects could not be attributed to the physical activity associated with learning outdoors (Dettweiler et al., 2017).

Not only is contact with nature tied to important factors in classroom engagement, but greener schools and classrooms have been tied to better academic achievement. Multi-year assessments of greenness around Massachusetts public schools found positive correlations between greenness and standardized test scores, even after adjusting for income and other confounding factors, although not for all seasons of the year (Wu et al., 2014). Similarly, standardized test performance in 3rd through 9th graders was higher for District of Columbia public schoolyards with higher levels of tree cover, even after similar controls (Kweon et al., 2017), and high school graduation rates and test scores were better for public high schools across Michigan with views of greenspace from high school classrooms and cafeterias (Matsuoka, 2010). More recently, standardized test scores have been tied to schoolyard tree cover in over 300 public schools in Chicago, again controlling for socioeconomic and other factors (Kuo et al., in review). While these studies do not directly connect nature exposure with increased classroom engagement, they are consistent with this possibility; indeed, it is difficult to imagine how contact with nature could boost academic achievement while reducing classroom engagement.

Thus, exposure to nature has been tied to both the antecedents and the consequences of classroom engagement. Additional converging evidence comes from research in educational psychology not focused specifically on greenness. Generally speaking, time spent out of the classroom and in relatively natural outdoor settings is positive. Studies document (a) the rejuvenating effects of recess (e.g., Pellegrini and Davis, 1993; Pellegrini et al., 1995; Jarrett et al., 1998), (b) the positive impacts of students' physical activity—often in schoolyards—on on-task behavior and executive functioning in the classroom (Mahar, 2011; Kvalø et al., 2017), and (c) the motivational benefits of teacher-led education outside the classroom (EotC)—in schoolyards, museums, and other cultural institutions (Dettweiler et al., 2015; for review see Becker et al., 2017) and of garden-based learning (Skinner et al., 2012). All these lines of investigation lend indirect support for the hypothesis that lessons in nature might enhance subsequent classroom engagement.

At the same time, it must be acknowledged that the question here differs importantly from those lines of investigation. This study differs from the research on the benefits of recess and physical activity in that the intervention involves formal instruction—teacher-led, formal lessons, delivered as part of a larger curriculum, with all the rules against student socializing and autonomous activity typical of classroom-based lessons. Similarly, unlike most education outside the classroom (EotC) studies and the study of garden-based learning, this study holds pedagogical approach constant in comparing lessons in nature vs. in the classroom. That is, in most EotC studies, the instruction outside the classroom is designed to take advantage of the setting; as a consequence, the experimental condition differs from the control in two ways—in setting (outside vs. in the classroom) and in pedagogical approach. In this study, pedagogical approach was held constant across conditions; the lessons inside and outside the classroom differed in setting but not instructional approach.

In sum, although it appears no study has directly examined the aftereffects of lessons in nature on classroom engagement, considerable evidence in both environmental psychology and education research points to time spent in natural outdoor settings as having positive impacts. In this study, we hypothesize that *lessons in nature have positive, immediate aftereffects on classroom engagement*—that is, we expect that when children learn outdoors, their classroom engagement after returning indoors is better than it would have been had they stayed inside the entire time. To test this hypothesis, we compared classroom engagement after a teacher gave her students a lesson in nature vs. after the same teacher gave her students a lesson on the same topic in the classroom (e.g., leaves) in the same week, replicating this comparison across 10 different topics (one topic per week), two classrooms (“classroom a,” with its own teacher, students, and room; and “classroom b,” with another teacher, set of students, and room), and five different measures of classroom engagement.

## Methods

### Setting and instructors

The effects of lessons in nature on subsequent classroom engagement were examined in the context of a 300-student environmental magnet school in the Midwestern United States serving a predominantly disadvantaged population, with 87% qualifying for free or reduced lunch, 82% African American, 7% Hispanic, 5% White, and 6% Multi-racial. Written consent from parents of involved students was obtained prior to the study.

**Figure 1:**
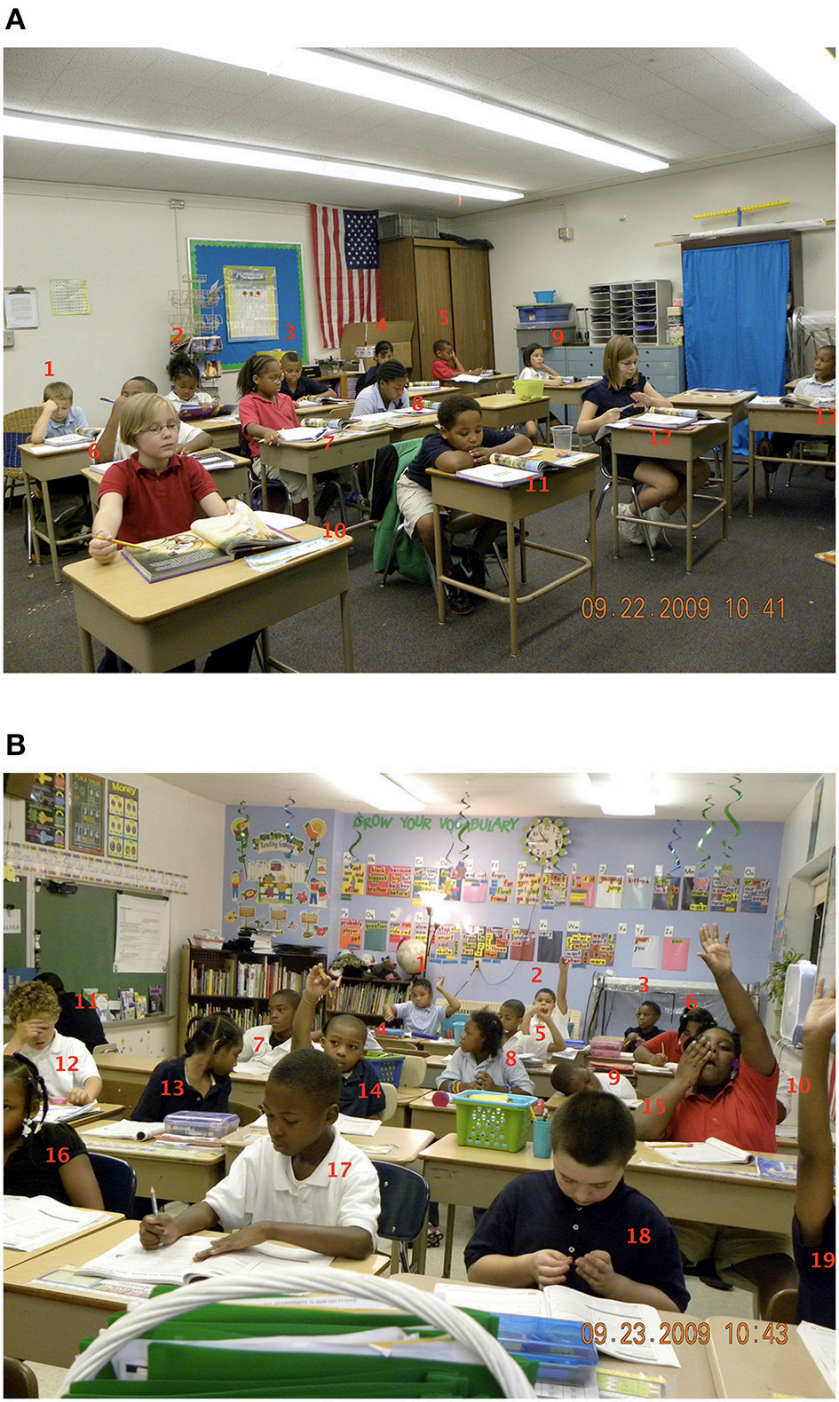
The two classrooms **(A,B)** used for indoor instruction in this study. Written permission for the publication of this figure was obtained from students' parents.

**Figure 2:**
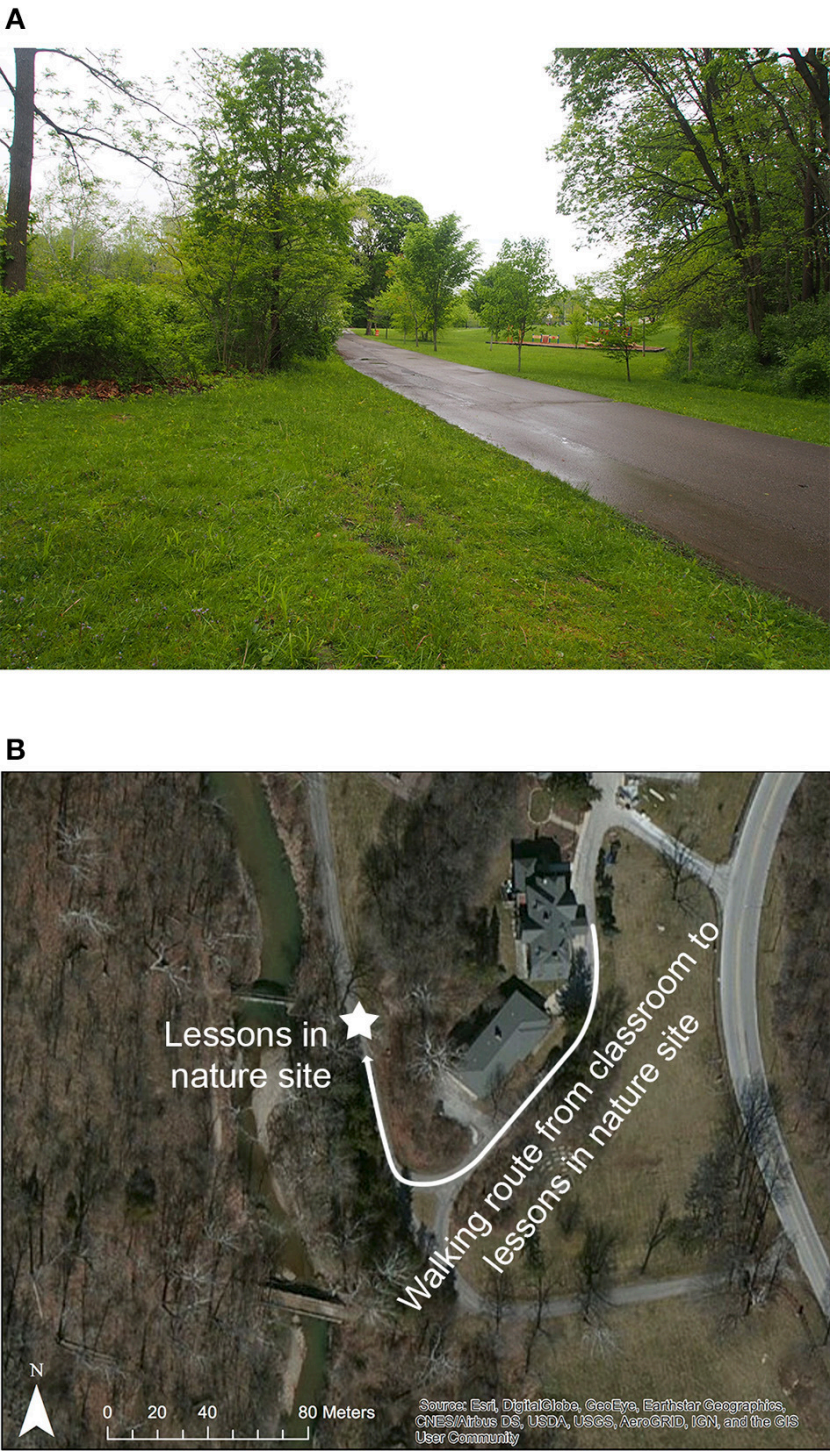
The site of the lessons in nature **(A)** and the route students took between their classroom and the outdoor lessons **(B)**. The road in the pictures was used exclusively for pedestrian traffic and (infrequently) for maintenance vehicles.

The indoor condition in this study comprised two typical classrooms (Figure 1; although they are not shown in the photo, both classrooms had windows). The outdoor condition comprised a small grassy area just outside the school (Figure 2). This instructional area was adjacent to a stream and woodlands, not used in the lesson. While the teacher was setting up the outdoor lesson, students occasionally visited the stream bank briefly. The post-treatment (and post-control) observation period was always conducted indoors, in each class' and teacher's regular classroom.

The two teachers in this study were highly experienced and state-certified in elementary education, with Masters in Education degrees and in-service training in outdoor and environmental education. These teachers had teamed together in lesson planning over a period of 5 years prior to this study, facilitating their coordination of lessons during this study.

The students in the classrooms were in third grade. Their age range was 9–10 years old.

### Design and procedure

At base, this study involved a mini-experiment replicated 20 times. In each mini-experiment, we examined classroom engagement after a lesson in nature vs. after a matched lesson in the classroom on the same topic, with the same teacher and students. Thus, in week 1 of our study, teacher “a” gave her students both a lesson on, say, leaf identification, outdoors, and another lesson on leaf identification in the classroom, and we compared indoor classroom engagement for that set of students after each of those two lessons. This mini-experiment was repeated across 10 different lesson topics and weeks (one topic per week), in each of two classrooms.

**Figure 3:**
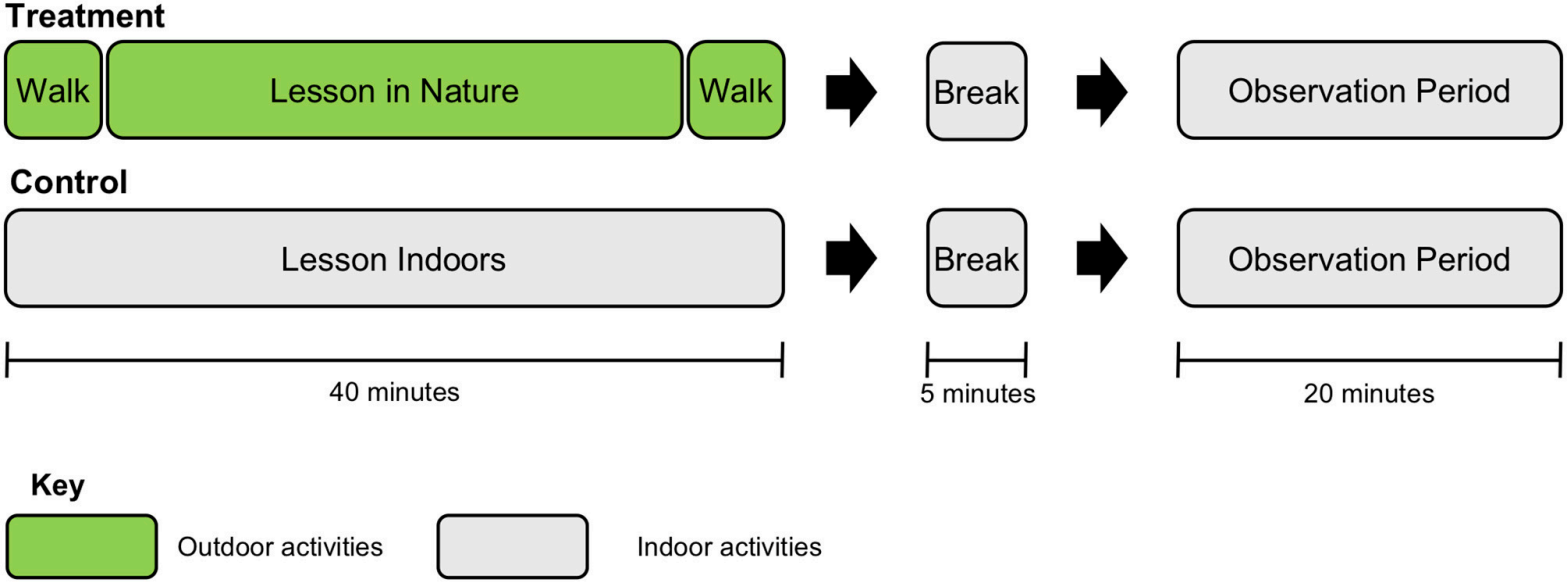
Schematic diagram of one mini-experiment. This included a treatment (lesson in nature and with walks to lesson site before and after) or a control (classroom lesson indoors), followed by a 5-min indoor break and 20-min indoor observation period. Order of conditions was counterbalanced.

Figure 3 schematically depicts a mini-experiment—the fundamental unit of comparison in this study. Both the experimental condition (the lesson in nature) and the control condition (the lesson in the classroom) were 40 min long, and the observation period for both conditions was 20 min long. Observation periods took place in the teacher's regular classroom, and included an introductory 5-min presentation by the teacher on math or language arts using a dry erase board, overhead projector, or chalkboard and 15 min of assigned individual student work completed at their desks. Before the observation period there was a water and bathroom break in both conditions.

**Figure 4:**
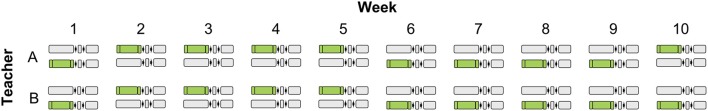
Mini-experiments were replicated over 10 different topics and weeks, for each of two classrooms (and each of five measures). Order of conditions was counterbalanced.

Figure 4 shows how we replicated our fundamental unit of comparison across different instructional content, times in the school year, students, classrooms, and instructors. Each pair of lessons (one in nature, one in the classroom) was delivered in a single week. For each pair, the two teachers worked together to adapt a different theme from the Project Learning Tree (www.plt.org) environmental education lesson guide, with lessons on leaf, tree, and seed identification; organic matter decomposition; the life cycle; and pollution. These two instructors each delivered 10 pairs of lessons over 10 different weeks in the semester from September-November, under a range of weather conditions^1^. Before the study began, both instructors were open-minded as to what we might find, although one tended to think the positive effects of lessons in nature might outweigh the negative, whereas the other tended to think the opposite—that lessons in nature might leave students “too wired” afterward to engage in classroom material.

To make the lessons as comparable as possible, each lesson pair was carefully matched along numerous dimensions. In addition, where exact matching was not possible we counterbalanced across the study so there were no consistent differences between conditions. For one notable dimension, neither matching nor counterbalancing was possible.

Lessons were matched along the following dimensions: teacher, students and class size, topic, teaching style, week of the semester, and time of day. That is, for any given pair of lessons, both the treatment lesson (in nature) and its indoor counterpart were delivered by the same teacher to the same students, on the same topic, in the same week of the semester. Both lessons involved hands-on, experiential learning; lessons that required natural materials from the outdoor instructional site (e.g., different types of leaves) were adapted for classroom instruction by bringing these materials indoors prior to the lesson. While the pairs of lessons were offered in afternoons (*n* = 12) slightly more often than in mornings (*n* = 8), the two conditions did not differ in how often they were taught in the morning vs. the afternoon—an important consideration given that cognitive performance generally drops over the course of the day (Sievertsen et al., 2016).

We counterbalanced the order in which conditions were delivered each week over the course of the study. It is impossible to offer both a lesson in nature and its matched classroom lesson simultaneously; thus one lesson would have to precede the other and the second lesson would always be an extension of the first. So that neither condition would have an advantage over the other, we encouraged teachers to put the lesson in nature first roughly as often as they put it second. The scheduling of lessons was constrained by the scheduling of other curriculum (e.g., physical education, art, and music) as well as weather. In the end, the lesson in nature came before its classroom counterpart four times and after it six times for each teacher.

It is important to note that there was one consistent difference between the experimental and control lessons other than setting. The 40-min lesson in nature was not purely instructional time; it required the class to walk a few minutes to and from a grassy area (see Setting above) to reach the instructional site—a distance of about 200 m. Thus, the lesson delivered in nature was roughly 30 min long whereas the matched indoor lesson was 40 min long.

### Measures of classroom engagement

We developed a battery of four measures to assess classroom engagement: (1) teacher ratings; (2) student ratings; (3) “redirects”—the number of times instructors had to interrupt instruction to redirect a student's attention to the task at-hand; and (4) independent photo ratings—ratings of classroom engagement by an independent observer based on photographs of the observation period. These four measures were then combined into a Composite Index of Classroom Engagement.

#### Teacher ratings

At the end of each 20-min observation period, teachers rated classroom engagement on a −2 to +2 scale (from −2 *much worse than usual* to 2 *much better than usual*, with 0 *same as usual*). Classroom engagement was defined for teachers as students listening to instructions, looking at assigned material, and raising their hands for assistance. Teachers were asked to rate the engagement not of individual students, but of the classroom as a whole, during the observation period.

#### Student ratings

Students also rated classroom engagement after each 20-min observation period. Unlike the teacher ratings, the student ratings consisted of three components. Each student rated their own engagement, the engagement of the students sitting close to them, and the engagement of the class as a whole on a 5-point scale indicating the period of engagement (from 1 *no time* to 5 *the whole time*).

Of the three types of engagement ratings—self, peer, and whole class—one turned out to be relatively uninformative and was not further analyzed: students consistently rated their own engagement highly and with little variance; perhaps as a consequence, this rating correlated relatively weakly with other measures (see Supplementary Materials). Students' ratings of the engagement of their seatmates and the class as a whole were somewhat informative in that they were not at ceiling and showed some variance; students' peer and whole class ratings were therefore used as another measure of classroom engagement. For each classroom after a given lesson, students' peer engagement ratings and whole class engagement ratings were averaged to produce an average, student-based measure of classroom engagement. This summary student-based measure of classroom engagement demonstrated high internal reliability (Cronbach's alpha = 0.869 for indoor lessons, 0.807 for outdoor lessons).

#### “redirects”

Each time a teacher needed to stop instruction to redirect or correct student behavior—e.g., “sit down,” “you need to be working,” or “I will wait”—one “redirect” was tallied. “Redirects” reflect the number of instances tallied for a 20-min observation period. Redirects are a concrete and important indicator of how well instruction is going. High levels of redirects indicate students are not attentive to instruction or tasks assigned. Further, redirects themselves are likely to impact learning outcomes by reducing the coherence and flow of lectures and distracting students as they work on assigned tasks.

MP, an investigator on this project and the social worker for the school where this study was conducted, was stationed at the back of the classroom during observation periods to record “redirects.” As the school social worker, the instructors and students in this study were already comfortable with his presence in the classroom. Pilot testing confirmed that he was able to observe the class from the back of the room without influencing class dynamics. Redirects were tallied “blind to condition”—that is, the observer assessed redirects without knowing whether the preceding lesson had been given indoors or outdoors.

#### Independent photo ratings

While teacher ratings and student ratings each provide a valuable window onto class engagement, both are inevitably subject to observer expectancy effects. That is, both teacher and student ratings of classroom engagement during a given observation period might be influenced by their knowledge of which condition (lesson in nature or lesson in the classroom) preceded that observation period and their expectations for the effects of lessons in nature on classroom engagement. Redirects were blind to condition, but we included a second “blind to condition” measure of classroom engagement, in which an independent observer rated photographs of each observation period without knowing what kind of lesson had preceded it.

Photographs were captured with a wide-angled camera (Nikon P90) positioned on a tripod in front of the classroom and programmed to automatically capture images of the class throughout the 20-min observation period. Each observation period was represented by 10 photos; hence the complete collection of photos rated by our independent observer consisted of 400 photos, with each set of 10 photos corresponding to one of the 40 observation periods in this study (one observation period per week after the lesson in nature, another observation period per week after its classroom-based counterpart, for each of two teachers, for a total of 10 weeks).

Our independent observer—an undergraduate student at the University of Illinois at Urbana-Champaign—began by acquainting herself with the entire collection of 400 photos, without knowing which observation periods belonged to which condition. This allowed her to calibrate her ratings of classroom engagement relative to both the typical levels of engagement seen in the observation periods as well as the extremes. She then rated classroom engagement for each observation period on the same −2 to +2 scale as the teachers (from −2 *much worse than usual* to 2 *much better than usual*, with 0 *same as usual*). The rater assessed classroom engagement blind to condition; that is, she made her ratings without knowing where the preceding lesson had taken place (in nature vs. the classroom).

#### Constructing a composite index of classroom engagement (CICE)

Each of the component measures in our battery is valuable in its own right. Teacher ratings and student ratings offer important lenses on classroom engagement. Redirects, as counted by an independent observer, provide external validation for teacher and student-ratings as well as a concrete measure of classroom engagement. Both redirects and the independent photo ratings provide measures of classroom engagement uncontaminated by knowledge of condition. Table 1 illustrates how each of the measures in our battery address different methodological criteria for assessing classroom engagement. Together, the measures in this battery provide a multifaceted measure of classroom engagement, with the limitations of each measure countered by the strengths of another.

**Table 1:** Measures and criteria for assessing classroom engagement.

**Measure**	**CRITERIA FOR ASSESSING CLASSROOM ENGAGEMENT**			
	**Incorporates teacher perceptions**	**Incorporates student perceptions**	**Provides external validation**	**Is blind to condition**
Teacher ratings	Yes	–	–	–
Student ratings	–	Yes	–	–
Redirects	–	–	Yes	Yes
Independent photo ratings	–	–	–	Yes
Composite index of classroom engagement	Yes	Yes	Yes	Moderately^a^

a *Two of four components of Index are blind to condition*.

To create a single measure that draws on each of these different methodological strengths, we combined these component measures into a single Composite Index of Classroom Engagement (CICE), which was the average of teacher ratings, student ratings, independent photo ratings, and redirects. Because these measures are on different scales (e.g., from −2 to +2 for teacher and photo-based ratings, from 0 to 100 for student ratings), data from each measure were standardized before averaging. Thus, for example, a teacher's rating of classroom engagement for a given observation period would be expressed in terms of how that period's rating differed from the mean rating for that teacher across all observation periods, in units of standard deviations. Redirects were reverse-coded (multiplied by −1.0) so that higher values would correspond to better classroom engagement, in line with the other components of the Composite Index.

## Results

### Descriptive statistics and bivariate correlations

Descriptive statistics and bivariate correlations are presented in Tables 2, 3. Teacher ratings of class engagement tended toward the positive, with average ratings falling between 0 *usual* and 1 *better than usual*. Student ratings of class engagement were quite positive, averaging roughly 80% on a 0–100% scale, with little variance. Redirects occurred with some frequency, averaging 3.7 and 5.1 in the two classrooms, respectively, in the 20-min observation window. And photo-based ratings of class engagement also tended toward the positive, with average ratings falling between 0 *usual* and 1 *better than usual*. As the CICE (Composite Index of Classroom Engagement) is based on the average of standardized scores across the four component measures for each classroom, its means for each classroom were zero by definition. In two-sided *t-*tests for group differences with an alpha of 0.05, the two classrooms did not significantly differ from each other on any of the measures of classroom engagement; thus data from the two classrooms were combined for further analysis except where otherwise noted.

**Table 2:** Means of classroom engagement measures by classroom.

		**Classroom A**		**Classroom B**	
	**Range**	***M***	***SD***	***M***	***SD***
Teacher ratings (−2–+2)	−2–2	0.70	1.34	0.55	1.23
Student ratings (0–100)	62–93	81.29	8.09	79.00	7.55
Redirects (tallied)	0–8	3.70	2.62	5.10	1.86
Independent photo ratings (−2–+2)	−2–2	0.35	1.42	0.65	0.99
Composite index of classroom engagement	−1.60–1.17	0.00	0.81	0.00	0.77

**Table 3:** Bivariate correlations between measures of classroom engagement across 40 observation periods.

	**1**	**2**	**3**	**4**	**5**
Teacher ratings (1)	–	0.48^**^	0.54^**^	0.87^**^	0.92^**^
Student ratings (2)		–	0.25	0.32^*^	0.63^**^
Redirect (3)			–	0.51^**^	0.70^**^
Independent photo ratings (4)				–	0.86^**^
Composite index of classroom engagement (5)					–

* *p < 0.5*,

** *p < 0.01*.

As Table 3 shows, our measures of classroom engagement were generally highly correlated. The individual components of the CICE show high concurrent validity. Teacher ratings and independent photo-based ratings were particularly highly correlated with both each other (*r* = 0.87) and with our summary measure (*r* = 0.92). Student ratings of classroom engagement were significantly correlated with teacher ratings and independent photo-based ratings, but not significantly related to the number of redirects in a given observation period.

### Overall condition differences in classroom engagement

Is classroom engagement higher after a lesson in nature than after a matched lesson in the classroom? Table 4 presents the results of paired, two-tailed *t-*tests comparing classroom engagement after lessons in nature vs. matched classroom lessons across the 10 different topics/weeks and two instructors. Lessons in nature show an advantage in subsequent classroom engagement over classroom lessons for four of the five measures. Teacher ratings of classroom engagement are roughly a standard deviation higher, on average, after a lesson in nature than its matched, classroom-based counterpart. Consistent with this, redirects were less frequent after a lesson in nature—in fact, the number of redirects after a lesson in nature was roughly half (54%) that of redirects after a classroom lesson. If we calculate the rate of redirects by dividing the duration of our observation period (20 min) by the number of redirects, the nature condition yielded a redirect rate of roughly one redirect per 6.5 min as compared to a rate of one interruption of instruction every 3.5 min in the classroom condition. The independent, photo-based ratings of classroom engagement echo the teacher ratings. And Composite Index of Classroom Engagement scores are 4/5ths of a standard deviation higher after lessons in nature than after matched control lessons. Effect sizes for all measures but the student ratings are substantial, indicating that the magnitude of the difference between classroom-based lessons and nature-based lessons is not only statistically significant but practically meaningful.

**Table 4:** Classroom engagement is better after lessons in nature than lessons in the classroom by most measures: Findings for each measure of classroom engagement.

	**Means**		**Paired differences**				
	**Nature**	**Classroom**	**Mean**	**Std. dev**.	***t-*value**	**df**	**Effect size^a^**
Teacher ratings	1.20	0.05	1.15	1.79	2.88^**^	19	0.74
Student ratings	81.01	79.27	1.74	6.56	1.18	19	0.60
Redirects	3.10	5.70	−2.60	2.62	4.43^***^	19	0.84^b^
Independent photo ratings	1.10	−0.10	1.20	1.64	3.27^**^	19	0.77
Composite index	0.40	−0.40	0.80	0.93	3.83^**^	19	0.81

a *Common language effect size (McGraw and Wong, 1992) also known as the probability of superiority (Grissom and Kim, 2005) expresses the effect size in percentages. In this table, it reflects the probability that the score for a given classroom engagement measure will be better after a lesson in nature than after a lesson in a classroom. Controlling for differences between classrooms in classroom engagement, the likelihood that a class will score higher on teacher ratings of classroom engagement after a lesson in nature than after a lesson in a classroom is 74%*.

b *For ease of interpretation, all effect sizes reflect the likelihood of better class engagement after a lesson in nature than a matched classroom lesson; because class engagement is better when redirects are fewer, the effect size reported here reflects the likelihood that redirects are fewer after a lesson in nature*.

** *p < 0.01*,

*** *p < 0.001*.

Bayesian statistical analyses yield similar results. The Bayes factor is a ratio of the likelihood of two hypotheses being correct given a set of data. In this case, we compared the likelihood that classroom engagement was better after outdoor lessons than after indoor lessons (H_1_) with the likelihood that it was not (H_0_). There was very strong evidence that the Composite Index of Classroom Engagement was better after outdoor lessons than after indoor lessons—so much so that H_1_ was 33 times more likely to occur than H_0_. In regard to individual measures, redirects showed extreme evidence for H_1_ occurring, indicating increased classroom engagement after outdoor lessons (BF_01_ = 0.009, error percent 8.07e^−7^), while independent photo-based ratings of classroom engagement displayed strong evidence (BF_01_ = 0.091, error percent = 5.12e^−4^) and teacher ratings of classroom engagement presented moderate evidence (BF_01_ = 0.18, error percent = 0.002) for this outdoor lesson advantage. In contrast, student ratings of classroom engagement showed no evidence of nature lessons improving classroom engagement afterward compared with indoor lessons (BF_01_ = 2.33, error percent = 0.014).

### Condition differences in classroom engagement for different classrooms, weeks, and measures

**Figure 5:**
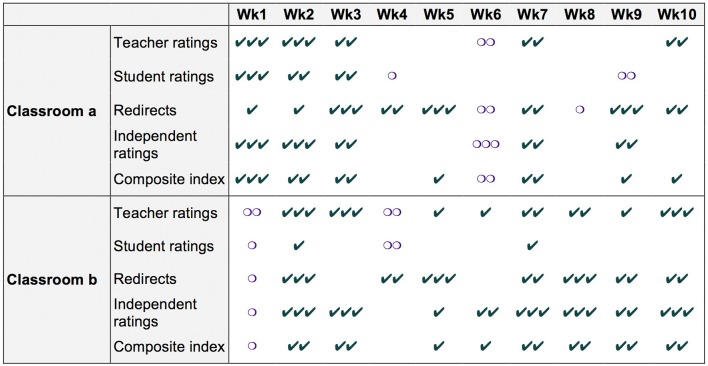
Differences in classroom engagement after lessons in nature for different classrooms, weeks, and measures. Condition differences in classroom engagement are depicted with symbols. The color and shape denotes the condition which yielded better classroom engagement, for a particular measure, classroom, and week; when the lesson in nature outperformed its paired classroom lesson, there are green checkmark(s); when the lesson in the classroom outperformed its paired nature lesson, there are purple circle(s). The number of symbols (checkmark or circle) represents the extent to which one condition outperformed the other, with one symbol corresponding to a difference between half a standard deviation and a full standard deviation (>0.5 to ≤1), two symbols corresponding to a difference between one and two standard deviations (>1 to ≤2), and three symbols corresponding to a difference of over two standard deviations. When the difference between a lesson in nature vs. the classroom did not exceed half a standard deviation, no symbols are depicted.

Our research design involved 100 paired comparisons between lessons in nature vs. their matched, classroom-based counterparts across two different instructors, 10 different topics and weeks, and five different measures of classroom engagement. To give a more fine-grained view of our results, Figure 5 schematically depicts the results for each of the 100 pairs of comparisons. Symbols of different colors and shapes indicate which condition, if any, showed an advantage in subsequent classroom engagement in a given mini-experiment (green checkmark = lesson in nature; purple circle = lesson in the classroom), and the number of symbols indicate the extent of the advantage (no symbols = the conditions differed by less than half a standard deviation; one = the conditions differed by between 0.5 and ≤1 standard deviation; two = between 1 and ≤2 standard deviations; three = over 2 standard deviations).

Figure 5 thus illustrates the consistency and size of the nature advantage over the entire series of mini-experiments. Of the 100 nature vs. classroom comparisons, the majority of comparisons (61) show an advantage for the lesson in nature, 25 show small or no difference (less than half a standard deviation in either direction), and only 14 show an advantage for the classroom-based lesson. Further, the size of the nature advantage is considerable: in 48 comparisons, the lesson in nature yielded classroom engagement scores a full standard deviation larger than its classroom-based counterpart; in 20 of these 48, the nature advantage was more than two standard deviations.

Visual inspection for differences across measures suggests that, of the four component classroom engagement measures, teacher ratings, redirects, and independent (photo-based) ratings are reasonably sensitive. By contrast, student ratings appear to be a relatively insensitive measure, showing fewer and smaller condition differences than the other measures.

Similarly, visual inspection reveals no obvious trends in the size of the nature advantage over the course of the semester; consistent with this, a *post-hoc*, two-tailed independent *t-*test comparing the difference between CICE scores for the first 5 weeks of the semester with CICE scores for the next 5 weeks showed no significant difference, *t*_(18)_ = −0.26, *p* = 0.80 (*M* = 0.86, *SD* = 1.00 for the first 5 weeks; *M* = 0.74, *SD* = 0.91 for the next 5 weeks). Interestingly, although one of the two teachers entered with some skepticism regarding the effects of lessons in nature on subsequent classroom engagement, the nature advantage is visible in both instructors' classes. Paired, two-tailed *t-*tests for each classroom show a significant effect of condition on classroom engagement for each instructor [*t*_(9)_ = 2.27, *p* = 0.049, for classroom *a*; *t*_(9)_ = 3.07, *p* = 0.01, for classroom *b*]. Bayesian statistical analyses confirmed there was no evidence for the first 5 weeks being different than the next 5 weeks (BF_01_ = 2.41, error percent = 2.31e^−5^). Also, Bayes factors showed moderate evidence for classroom *a* (BF_01_ = 0.20, error percent = 3.41e^−4^) and anecdotal evidence for classroom *b* showing an outdoor lesson advantage (BF_01_ = 0.56, error percent = 0.002).

## Discussion

What is the effect of lessons in nature on subsequent classroom engagement? Do they leave pupils too keyed up to focus—as some teachers worry—or do they enhance a class' engagement—as indirect evidence suggests they could? In this study, classroom engagement was significantly better after lessons in nature than after matched, classroom-based lessons. This nature advantage held for four of five measures of classroom engagement: teacher ratings; redirects; independent, photo-based ratings; and our summary index of classroom engagement all showed a substantial advantage for the nature condition; student ratings did not. Further, the nature advantage held across different teachers and held equally over the initial and final 5 weeks of lessons.

The nature advantage was substantial. Common language effect size calculations (McGraw and Wong, 1992) indicate a strong advantage for lessons in nature—the likelihood that Composite Index of Classroom Engagement scores are higher after a lesson outdoors in nature than after a lesson in the classroom, in a class that receives both, is 81%. And the nature advantage is large. Out of 100 paired comparisons, classroom engagement was over a full standard deviation better in the nature condition in 48 pairs; in 20 of those 48, the nature condition bested its classroom counterpart by over two standard deviations. The rate of “redirects,” or instances where a teacher interrupted the flow of instruction to redirect students' attention, was cut almost in half after a lesson in nature. Normally, these redirects occur roughly once every 3.5 min of instruction; after a lesson in nature, classroom engagement is such that teachers are able to teach for 6.5 min, on average, without interruption.

### Accounting for the advantage of lessons in nature: alternative explanations

To what might we attribute the advantage of the lessons in nature here? There are any number of other factors that might affect classroom engagement: different teachers might be more skilled at eliciting student engagement; some topics are more engaging than others; hands-on lessons might be more engaging than lecture-based lessons; one set of students might be more attentive than another; a smaller class might be more engaged than one with more students; one classroom might be exposed to more distractions than another (for example, opening onto a particularly noisy hallway); engagement might peak at the beginning of the school year and flag as the year wore on; and students might find it easier to focus on schoolwork in the morning than the afternoon. If our nature lessons differed from our classroom lessons in any of these respects, those differences could have conceivably accounted for our findings. But because we only compared pairs of lessons *matched* on all those factors—same teacher, same topic, same instructional approach, etc.—none of those factors can account for the findings here.

Nor could positive expectations have driven the nature advantage here. It is true that one of the two teachers was predisposed to think the lesson in nature might have a positive effect on subsequent classroom engagement. Those positive expectations might have led her to view classroom engagement after the outdoor lesson more positively (which might have boosted teacher ratings of engagement but would not have affected our independent photo-based ratings), or might even, in a variant of the Pygmalion effect, have inspired her to teach more effectively afterwards (which would have boosted both teacher ratings and independent photo-based ratings). At the same time, the other teacher expected the opposite pattern; on the whole, she thought that the lesson in nature might leave students too keyed up to concentrate. If the nature advantage was due entirely to teacher expectations it is not clear why both teachers showed the nature advantage.

It should be noted that teacher expectations about the impacts of nature on subsequent classroom engagement may have become more positive over the course of the study, contributing to the nature advantage. However, this begs the question, why did teachers' expectations about the impacts of nature become more positive with experience if not because they had seen the positive impacts? Thus, a change in teacher expectations may well reflect, as well as contribute to, the nature advantage.

The novelty of the setting cannot account for the nature advantage, either. If the nature advantage in subsequent classroom engagement were due to the novelty of the setting, we would expect it to decrease over the course of the semester as students habituated to having lessons outdoors. But the nature advantage, as measured by the difference between nature-based lessons vs. classroom-based lessons in composite scores of classroom engagement afterward, was relatively stable over the course of the study. The nature advantage for the first 5 weeks of the semester and when the setting was relatively new was not statistically different from the nature advantage for the second 5 weeks—when students had acclimated to lessons outdoors.

Along similar lines, novelty of topic might have accounted for differences in classroom engagement; each week in the study corresponded to a new topic, and if the nature lesson on a topic had generally preceded its classroom counterpart, students might have found the nature lesson more stimulating and been more engaged afterwards because of the change in topic and not because of the setting. But the order of indoor and outdoor lessons was counterbalanced such that the lesson in nature came before its classroom counterpart four times and after it six times for each teacher.

In the absence of other viable explanations for the systematic pattern of superior classroom engagement after lessons in nature, it would appear that the lessons in nature boost subsequent classroom engagement.

### Accounting for the advantage of lessons in nature: active ingredients

If lessons in nature boost subsequent classroom engagement, this raises another question: what *about* lessons in nature might account for this effect? That is, what is (or are) the active ingredient(s) in a lesson in nature? Previous research suggests a number of possibilities; each of these factors might contribute. First, the *relatively natural setting* of the outdoor lessons may contribute to subsequent classroom engagement. As discussed in the Introduction, exposure to nature has immediate, beneficial aftereffects on both attention and stress, and is likely to enhance motivation as well. Further contact with nature has also been shown to improve self-discipline and impulse control (e.g., Faber Taylor et al., 2002; van den Berg and van den Berg, 2011)—thus a lesson in nature might conceivably yield a quieter, less disruptive classroom afterwards. It is interesting to note that the large effect sizes here were obtained despite the fact that the classrooms both had windows and therefore afforded some limited view of greenness. This has some precedent; previous findings have tied better outcomes for children's attention from being in nature than from simply looking at it (Faber Taylor et al., 2001).

Second, the sheer *break from classroom activity* involved in the walks to and from the classroom, and the *change in scenery* involved in the lesson in nature probably contribute to students' subsequent rejuvenation. Again, although this study involved formal instruction, not recess, Pellegrini and Davis (1993) and Pellegrini et al. (1995) found that elementary school children become progressively inattentive when recess is delayed. Another experimental study (Jarrett et al., 1998) found that fourth-graders were more on-task and less fidgety in the classroom on days when they had had recess, with hyperactive children among those who benefited the most. Thus, providing a lesson in nature may provide many of the same benefits normally accrued through recess.

The education outside the classroom (EotC) literature provides converging findings. Although EotC studies examine instruction not just in nature but also in museums and other settings outside the classroom, those studies all involve a change in scenery and some break from classroom activity to get to the alternate settings. Available evidence suggests that the social and learning outcomes of education outside the classroom are almost entirely positive (see Becker et al., 2017, for review). If a brief break from classroom activity and change of scenery suffice to deliver the improvements in subsequent classroom engagement seen here, teachers might experiment with simply taking their class to the gym for a lesson, or swapping classrooms with another teacher.

Similarly, the work on school garden-based learning suggests that student interest and motivation may improve when instruction is set outdoors in green areas, perhaps because of the greater autonomy and opportunities for social connection afforded by most garden-based curricula (Skinner et al., 2012; for a review of the role of autonomy and relatedness in motivation in educational settings, see Deci et al., 2011). While the findings here echo those, it is important to note that the lessons in nature here were formal and constructed to match those offered indoors; this was not informal learning but rather teacher-led, formal learning with the usual rules against students engaging in autonomous behavior or socializing—thus any effects of increased autonomy and relatedness would have to have occurred primarily in the walk to and from the outdoor lessons.

Third, *physical activity* might also play a part: 10-min physical activity breaks during the school day have been shown to boost classroom engagement (Mahar, 2011), and the lesson in nature here included two 5 min (or less) walks between the classroom and the outdoor teaching setting, raising the possibility that the boost in classroom engagement here was due entirely to those walks. This seems unlikely; most studies in the physical activity-classroom engagement literature have examined either brief bouts of intense physical activity (e.g., Mahar, 2011), or frequent, longer bouts of moderate physical activity—for example, one study examined the effects of adding roughly 190 min per week of moderate to vigorous physical activity—running, jump rope, hopping on one foot—over the course of 10 months (e.g., Kvalø et al., 2017). The dose of physical activity here was brief, light in intensity, and infrequent (two, 5 min walks per week). It seems likely that the physical activity involved in this study contributed to some but not all of the effects seen here.

Fourth and finally, another contributing factor may have been *impacts on teachers*. Teachers, just as much as students, might benefit from all these aspects of lessons in nature—perhaps teachers are able to teach in a more engaging way after a bit of walking, a bit of a breather and change in scenery, and a dose of nature has rejuvenated their attention and interest and reduced their stress levels. If so, simply giving teachers a break, a walk, and a dose of nature while their students continued formal instruction might yield the same benefits to classroom engagement seen here.

Each of these active ingredients has, in theory, the potential to singly explain the effect of lessons in nature on classroom engagement. Given the size of the nature advantage found here, it seems likely that the effect reflects the joint impact of all these factors.

### Generalizability

The lessons in nature here involved a particular “dose” (duration, intensity, and frequency) of naturalness, administered in a particular way, to a particular population of students by a particular set of teachers. Here, we consider reasons why the nature advantage might or might not generalize to other conditions, students, and teachers.

The lessons in nature in this study involved a 5-min walk from the classroom out to a grassy outdoor area with some nearby trees (Figure 2) for a 30-min instructional period, followed by a walk back to the classroom, followed by a 5-min break—the classroom lesson involved no walking, and a 40-min instructional period followed by a 5-min break.

In combination with the study design, the findings here suggest the nature advantage could apply in a variety of conditions. The nature advantage persisted across 10 different topics and weeks in the school year; across different times of day; across two different teachers, including one who was predisposed to expect the opposite; and across two different groups of students, each with their own dynamics.

The levels of vegetation here (Figure 2) do not seem entirely out of keeping with other schools; schools with similar levels of vegetation within walking distance might reasonably expect similar effects to those here. But many urban schools might have more barren schoolyards and surrounds—in those schools, we might still expect an advantage for lessons outdoors if the environment is reasonably safe, as some evidence suggests that outdoor settings without vegetation have effects better than indoor settings although not as good as green outdoor settings (Kuo and Faber Taylor, 2004). In schools with considerably greener surrounds, lessons in nature might have even larger impacts on classroom engagement; in one of the few studies including a wide of levels of nearby nature, the more natural a students' dormitory view, the better their cognitive performance (Tennessen and Cimprich, 1995).

The students in this study were predominantly low-income, students of color. In students facing challenges associated with poverty, minority status, or both, academic achievement is a pressing concern—in a comparison of rich and poor school districts, sixth graders in the richest school districts are four grade levels ahead of children in the poorest districts, and differences in socioeconomic status explain much but not all racial/ethnic differences in outcomes (Reardon et al., 2016). In this population, then, the finding of an inexpensive educational practice with a consistent, large, positive effect on classroom engagement raises exciting possibilities. As for other populations, the available evidence suggests that similar effects might obtain: in the greenspace-academic achievement literature (e.g., Matsuoka, 2010; Wu et al., 2014), schools with lower numbers of free-lunch eligible students and non-Whites show positive relations between nearby greenspace and standardized test scores.

The teachers in the study were both highly experienced, had had in-service training in outdoor and environmental education, and were open-minded as to what the study might reveal. It seems plausible that teachers without such training, and teachers adamantly opposed to lessons in nature, might show smaller effects or even none at all. Their relevant in-service training is likely to have given the teachers more confidence in offering lessons in nature, and as highly experienced instructors, they may have been more adept at recognizing the need for adjustments and making them. Thus, the effects found here might reflect these teachers' background in outdoor and environmental education. At the same time, teachers with their background might well be precisely the population of teachers most ready and willing to try offering lessons in nature.

### Contributions to the science of nature-based learning

The findings here fill a gap in the previous literature on the impacts of nature on human functioning. On the one hand, previous experimental work has shown immediate aftereffects of contact with nature on basic psychological processes relevant to classroom engagement—attention, intrinsic interest in learning, impulse control, stress, and the effects of physical activity on cognitive functioning. On the other, large-scale correlational work has tied greener near-school landscapes with better school-level performance on standardized academic achievement tests—even after controlling for socioeconomic and other factors. These two lines of investigation examine different kinds of functioning, scales of analysis, and units of time. The work here bridges the two lines of investigation, pointing to a potential pathway between the two.

Boosts in classroom engagement might be a steppingstone by which nature's immediate, short-term effects on basic psychological processes might ultimately translate into boosts in long-term academic outcomes at the school level. Boosting attention, intrinsic motivation, and discipline simultaneously while reducing stress within the same individual seems likely to have synergistic effects in student-level engagement. Across pupils in the same class, boosting engagement in multiple students simultaneously is likely to result in synergies as well; when many, if not all, of the students in a class are quieter, more focused and less disruptive, classroom engagement is likely to be much fuller and more sustained. These two synergies—between different psychological processes within individual students, and between students within a class—may explain the size of the nature advantage seen here at the classroom level. Furthermore, because classroom engagement is an important contributor to long-term academic achievement (Skinner and Belmont, 1993; Godwin et al., 2016), regular episodes of exceptional classroom engagement over the course of a school year might have a surprisingly large cumulative effect on learning. Theoretically, this may help explain how relatively small differences in near-school green cover have been tied to significant differences in end-of-year standardized test performance (Matsuoka, 2010; Wu et al., 2014; Hodson and Sander, 2017; Kweon et al., 2017; Browning et al., in review; Kuo et al., in review).

For scientists interested in examining the impacts of lessons in nature on classroom engagement—or, more generally, following changes in classroom engagement over time—the Composite Index of Classroom Engagement and its constituent components may be of use. The CICE differs from other measures of engagement in two ways. First, it focuses on engagement at the level of the classroom rather than the individual student (for a review of 21 measures of individual student-level engagement, see Fredricks et al., 2011). And second, our measure is designed to provide a global assessment of classroom engagement for a class within a specified time window, and to allow tracking changes within a class over time. By contrast, the similarly titled “Classroom Engagement Inventory” (CEI) (Wang et al., 2014) was designed to quantify differences between classrooms in classroom engagement. Although our CICE can also be used to compare different classrooms, it does not separately assess the affective, behavioral, and cognitive dimensions of engagement as the CEI does; however the CICE does have the advantage of incorporating teacher's perceptions without relying entirely on teacher report.

We recommend future researchers use the measures showing the highest concurrent validity and sensitivity to the intervention here: teacher ratings, redirects, and independent photo-based ratings, and a composite measure. Although student-based ratings of classroom engagement—or more specifically student ratings of peer engagement and whole class engagement—had reasonable levels of interrater reliability and correlated positively with other measures of engagement, they were not sensitive to condition differences in engagement and may not be worth the trouble of collecting. Teacher ratings, by contrast, are quickly and easily collected, and seem an invaluable source of data as they reflect teachers' self-reflections on how easy or difficult students were to engage. Redirects—instances in which the instructor stopped instruction to redirect or correct student behavior, “sit down,” or “I will wait”—are a concrete and important indicator of how well instruction is going. High levels of redirects indicate students are not attentive to instruction or tasks assigned. Further, redirects themselves are likely to impact learning outcomes by reducing the coherence and flow of lectures and distracting students as they work on assigned tasks. And the use of photo-based independent ratings allows ratings of classroom engagement to be made blind to condition and outside of the teacher's perceptions or biases, without having to introduce an experimenter in the classroom.

### Implications for educational practice

The findings here provide some support and guidance for including more lessons in nature in formal education. For teachers who have been intrigued by the potential of lessons in nature but have been concerned about negative aftereffects on classroom engagement, the findings here directly address that concern. For environmental educators who have been shunted aside in favor of spending instructional time on drill and practice for standardized achievement tests, the findings here may offer a valuable argument for outdoor environmental lessons. The findings here also offer some encouragement for teachers interested in trying to adopt experiential approaches to education, which are particularly well-suited for lessons in nature. Such approaches allow students to actively use the outdoors to apply theoretical knowledge “in the field” and undertake problem-solving and decision-making in real world scenarios. These processes may be more effective at instilling and scaffolding long-term knowledge acquisition than other instructional strategies (Ballantyne and Packer, 2002). Curriculum that could benefit from learning styles beyond auditory and visual are also particularly well-suited for lessons in nature, because the diversity of topography and vegetation in natural landscapes also provide unique kinesthetic learning opportunities (Fjørtoft and Sageie, 2000; Auer, 2008).

While we do not know to what situations and populations the effects here will generalize, the consistency and size of the effects here suggest that lessons in nature are worth trying in a broad range of settings (for resources on how to start, see Supplementary Materials). It is worth noting that the nature advantage, while consistent, did not occur in every pair of lessons; notably, for one teacher the first classroom lesson outperformed its outdoor counterpart. Thus, we encourage teachers to try at least two or three lessons in nature before assessing their value.

More broadly, the findings here underscore the growing view that classroom engagement is at least as limited and valuable a resource as instructional time. With the advent of No Child Left Behind legislation, the vast majority of U.S. school administrators reduced or completely cut recess time and other breaks during the school day, with the primary motivation of providing more instructional time for standardized test preparation (Robert Wood Johnson Foundation, 2010). Instructional time has been viewed by many administrators as the key, limited resource for improving academic achievement; consequently, the de facto approach to increasing student learning has been to free up instructional time by cutting school activities seen to be unhelpful to standardized test preparation—recess, physical education, art, music, theater, etc. Yet increasing the number of hours in the classroom does not translate to increasing the number of hours of student are attentively learning (Gettinger and Seibert, 2002). Estimates suggest students spend 10–50% of their time at school *unengaged and off-task* (Hollowood et al., 1994). Like pouring tea into an already full teapot, giving teachers more time to deliver standardized test content is of little value if the vessels are unable to receive. Thus, classroom engagement may in fact be the key, limited resource in academic achievement. Seen in this light, the net benefits of recess, art, music, theater, and physical education for subsequent classroom engagement may easily exceed the tradeoff in instructional time—even apart from their inherent value.

### Priorities for future research

In our view, three tasks are pressing for future research: first, mapping the dose-response curve; second, assessing the net impact of lessons in nature for academic achievement; and third, establishing the generality of the effects here.

A map of the dose-response curve would be of great practical value. How “natural” does a landscape need to be to boost classroom engagement? If a small investment in vegetation outside a school can enable teachers to teach longer periods uninterrupted, such effects might ultimately translate to greater academic achievement in students, and more job satisfaction and less burnout among teachers. Similarly, studying larger doses than those here may reveal even larger benefits. The fact that the effect of each outdoor lesson does not diminish even as such lessons become routine suggests that adding more, or longer, lessons might yield proportionately large benefits. Perhaps instead of going out for lessons once a week, students might go out once or twice a day. Similarly, more prolonged or more intense doses of nature might be worth testing, such as is typical in “all-weather schools” or “outdoor schools” in Europe (Bentsen and Jensen, 2012). The larger landscape of the school in this study included a fishing stream and 30 acres of woodlands and open space that might theoretically be resources for lessons in nature, but the teachers in this study were reluctant to sacrifice the necessary instructional time to walk to those areas. The findings here suggest that the benefits of such larger doses of nature might be well worth investigating.

In addition to mapping the dose-response curve, there is a pressing need to quantify the net impact of lessons in nature on academic achievement. Substantial evidence points to lessons in nature enhancing learning of the material in those lessons; to what extent do lessons in nature enhance learning of the material in subsequent lessons? What is the net effect on academic achievement, given that some instructional time is spent on walking to and from lesson sin nature? The large effects here on classroom engagement suggest potentially large boosts in academic achievement.

A third priority for research should be to establish the generality of the effects here. The success of this intervention in two real-world classrooms over a variety of lessons, weather conditions, and initial teacher expectations invites expanded testing. Does it matter what the subject of the lesson in nature was? In this study, the topics all fell within the general domain of biology. Might a poetry class held outdoors have similar effects? Similarly, the teachers here were experienced and highly trained; might less seasoned instructors have less success managing an outdoor class? Further, in this study the students came from largely disadvantaged, urban neighborhoods; to the extent that these populations might experience less contact with nature than others, perhaps the impact of even small doses of nature is heightened. Future research on the aftereffects of lessons in nature should incorporate students from less urban, less disadvantaged contexts, as well.

## Conclusion

This study is the first to our knowledge to directly examine the effects of lessons in nature on subsequent classroom engagement. We found higher levels of classroom engagement after lessons in nature than after carefully matched classroom-based counterparts; these differences could not be explained by differences in teacher, instructional approach, class (students, classroom, and class size), time of year, or time of day, nor the order of the indoor and outdoor lessons on a given topic. It would seem that lessons in nature boost subsequent classroom engagement, and boost it a great deal; after a lesson in nature, teachers were able to teach for almost twice as long without having to interrupt instruction to redirect students' attention. This nature advantage persisted across 10 different weeks and lesson topics, and held not only for a teacher with positive expectations for nature-based lessons but also for a teacher who anticipated negative effects of such lessons. The findings here suggest that lessons in nature allow students to simultaneously learn classroom curriculum while rejuvenating their capacity for learning, or “refuel in flight.” Because providing children with more contact with nature in the course of the school day is likely to yield a whole host of additional dividends as well, including improved physical and mental health (see Chawla, 2015 for review), the findings here argue for including more lessons in nature in formal education.

## Ethics statement

This study was carried out in accordance with the recommendations for the Protection of Human Subjections, Institution Review Board, University of Illinois at Urbana-Champaign. Parents gave written informed consent in accordance with the Declaration of Helsinki. The protocol was approved by both the University of Illinois at Urbana Champaign Institutional Review Board and the Indianapolis Public Schools Department of Research, Evaluation and Assessment.

## Author contributions

MK was involved in study design, the development of measures, data acquisition, data analysis, and manuscript writing. MB was involved in data analysis and manuscript writing. MP was involved in the study design, the development of measures, data acquisition, and data analysis, and commented on the manuscript.

### Conflict of interest statement

The authors declare that the research was conducted in the absence of any commercial or financial relationships that could be construed as a potential conflict of interest.

## Footnotes

^1^On one occasion, a planned lesson was not given as scheduled; that lesson was made up in April instead. Analyses with and without the makeup lesson and its paired classroom lesson show the same effects of lessons in nature on subsequent classroom engagement. Findings reported here were based on the full sample.
