# Supplementary material for: Do Lessons in Nature Boost Subsequent Classroom Engagement? Refueling Students in Flight
Source: Front Psychol. 2018 Jan 4;8:2253. doi: 10.3389/fpsyg.2017.02253 (PMC5758746; doi:10.3389/fpsyg.2017.02253)
Supplement: Supplementary file 1 [file Presentation1.PDF]

Supplementary Material

Do Lessons in Nature Boost Subsequent Classroom Engagement? Refueling Students in Flight

Ming Kuo*, Matthew H. E. M. Browning, Milbert L. Penner

*** Correspondence:** Corresponding Author: fekuo@illinois.edu

Supplementary Presentation

Resources for Educators: Richard Louv's blog provides 10 succinct steps for teachers to begin to use nature for learning (http://richardlouv.com/blog/every-teacher-can-be-a-natural-teacher/). A network of teachers share curriculum strategies and success through the Natural Teachers Network (http://childrenandnature.ning.com/group/naturalteachers). Creative STAR Inc. provides many free lessons in nature resources on their website (http://creativestarlearning.co.uk). And the Arbor Day Foundation hosts a series of trainings and workshops for teachers and school administrators to build safe natural areas that promote learning (https://natureexplore.org).
