# Supplementary material for: Do Lessons in Nature Boost Subsequent Classroom Engagement? Refueling Students in Flight
Source: Front Psychol. 2018 Jan 4;8:2253. doi: 10.3389/fpsyg.2017.02253 (PMC5758746; doi:10.3389/fpsyg.2017.02253)
Supplement: Supplementary file 2 [file Table1.DOCX]

Supplementary Material

Do Lessons in Nature Boost Subsequent Classroom Engagement? Refueling Students in Flight

Ming Kuo*, Matthew H. E. M. Browning, Milbert L. Penner

*** Correspondence:** Corresponding Author: [fekuo@illinois.edu](mailto:fekuo@illinois.edu)

# Supplementary Table 1

Means of three types of student ratings

|  |  | **Classroom A** | | **Classroom B** | |
| --- | --- | --- | --- | --- | --- |
|  | Range | M | SD | M | SD |
| Student ratings of themselves | 81-99 | 93.00 | 3.74 | 90.59 | 4.79 |
| Student ratings of classmates sitting next to them | 55-94 | 80.77 | 9.32 | 78.64 | 9.61 |
| Student ratings of the class as a whole | 67-94 | 81.81 | 7.65 | 79.36 | 6.80 |

**
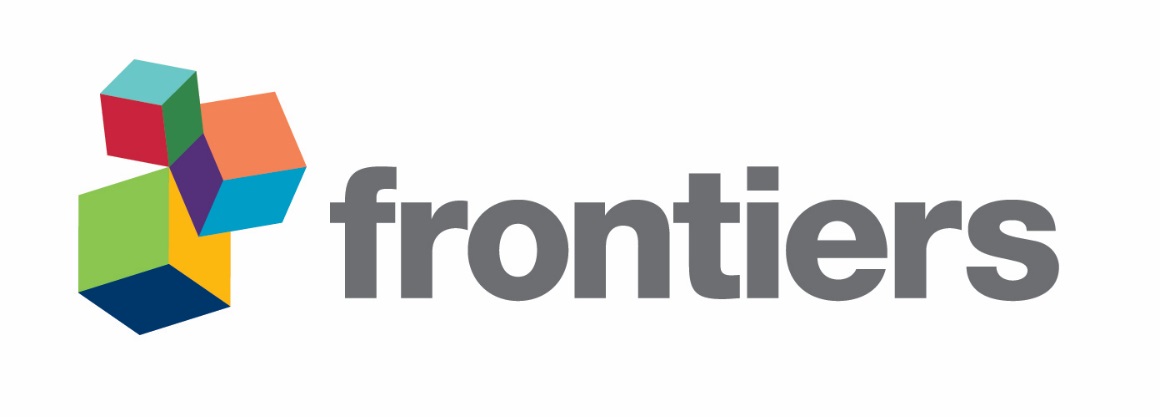
**
