# Supplementary material for: Do Lessons in Nature Boost Subsequent Classroom Engagement? Refueling Students in Flight
Source: Front Psychol. 2018 Jan 4;8:2253. doi: 10.3389/fpsyg.2017.02253 (PMC5758746; doi:10.3389/fpsyg.2017.02253)
Supplement: Supplementary file 3 [file Table2.DOCX]

Supplementary Material

Do Lessons in Nature Boost Subsequent Classroom Engagement? Refueling Students in Flight

Ming Kuo*, Matthew H. E. M. Browning, Milbert L. Penner

*** Correspondence:** Corresponding Author: [fekuo@illinois.edu](mailto:fekuo@illinois.edu)

# Supplementary Table 2

Bivariate correlations between three types of student ratings

|  | 1 | 2 | 3 |
| --- | --- | --- | --- |
| Student ratings of themselves (1) | - | 0.61** | 0.46** |
| Student ratings of classmates sitting next to them (2) |  | - | 0.76** |
| Student ratings of the class as a whole (3) |  |  | - |

**
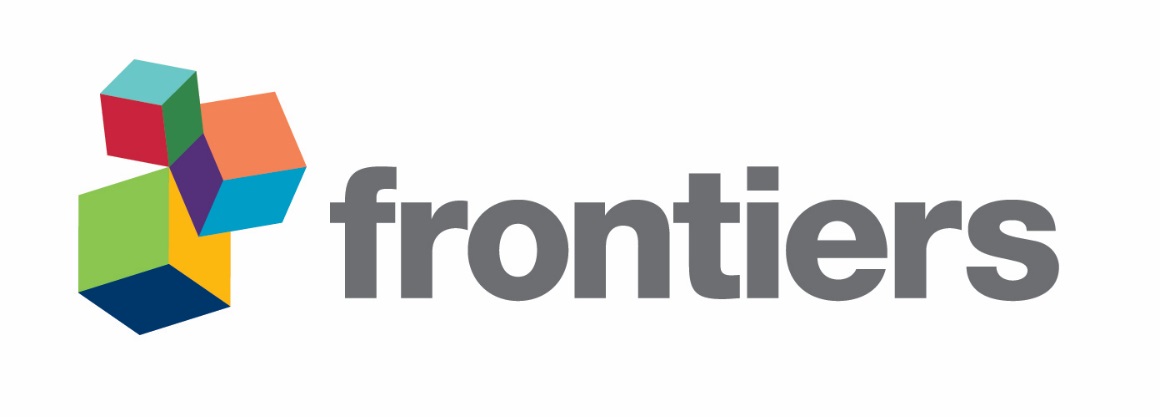
**
